# PET AIF estimation when available ROI data is impacted by dispersive and/or background effects

**DOI:** 10.1088/1361-6560/acc634

**Published:** 2023-04-07

**Authors:** Finbarr O’Sullivan

**Affiliations:** 1 Department of Statistics, University College Cork, Cork, Ireland

**Keywords:** PET, impulse response, regularization, constraints, arterial input function, spillover, dispersion

## Abstract

*Objective.* Blood pool region of interest (ROI) data extracted from the field of view of a PET scanner can be impacted by both dispersive and background effects. This circumstance compromises the ability to correctly extract the arterial input function (AIF) signal. The paper explores a novel approach to addressing this difficulty. *Approach.* The method involves representing the AIF in terms of the whole-body impulse response (IR) to the injection profile. Analysis of a collection/population of directly sampled arterial data sets allows the statistical behaviour of the tracer’s impulse response to be evaluated. It is proposed that this information be used to develop a penalty term for construction of a data-adaptive method of regularisation estimator of the AIF when dispersive and/or background effects maybe impacting the blood pool ROI data. *Main results.* Computational efficiency of the approach derives from the linearity of the impulse response representation of the AIF and the ability to substantially rely on quadratic programming techniques for numerical implementation. Data from eight different tracers, used in PET cancer imaging studies, are considered. Sample image-based AIF extractions for brain studies with: ^18^F-labeled fluoro-deoxyglucose and fluoro-thymidine (FLT), ^11^C-labeled carbon dioxide (CO2) and ^15^O-labeled water (H2O) are presented. Results are compared to the true AIF based on direct arterial sampling. Formal numerical simulations are used to evaluate the performance of the AIF extraction method when the ROI data has varying amounts of contamination, in comparison to a direct approach that ignores such effects. It is found that even with quite small amounts of contamination, the mean squared error of the regularised AIF is significantly better than the error associated with direct use of the ROI data. *Significance.* The proposed IR-based AIF extraction scheme offers a practical methodological approach for situations where the available image ROI data may be contaminated by background and/or dispersion effects.

## 1. Introduction

The emerging class of new PET scanners with long axial fields of view (FOV) (Badawi *et al* 2019, Pantel *et al* 2020, Alberts *et al* 2021), have given renewed impetus to the methodologies that can facilitate the creation of kinetic maps from dynamically acquired PET studies in humans. The time-course of the injected tracer in the arterial blood—the arterial input function (AIF)—is typically an essential input for kinetic analysis (Huang *et al* 1986, Wang *et al* 2020, Feng *et al* 2021, Gu *et al* 2021, Sari *et al* 2022). In cases where it is difficult/impractical to measure the AIF directly by blood sampling, it becomes necessary to recover the AIF from an analysis of the time-course for an image-derived region of interest (ROI) corresponding to an appropriate blood pool in the scanner FOV. In this context the importance of accounting for contamination in the ROI data, such as background spillover and/or dispersive effects, is well appreciated (Iida *et al* 1986, Gambhir *et al* 1989, Feng *et al* 1993). The work here is focused on this situation.

The representation of the AIF is a key part of any procedure used in image-based extraction of the AIF. Several parametric representations have been proposed, ranging from adjustable population templates (Olshen and O’Sullivan 1997, Christensen *et al* 2014, Rissanen *et al* 2015) to more sophisticated parametric model-based approaches (Huang *et al* 1991, Graham 1997, Huang and O’Sullivan 2014). The approach here uses a highly flexible non-parametric linear formulation in which the constraints needed to address limitations arising from spillover and/or dispersion can be simply addressed. The AIF is represented as a convolution of between the unknown whole-body impulse response to the tracer injection and the wave-form for the tracer injection actually delivered. The impulse response based representation gives the ability to separate the complexities of the injection profile structure when estimating the AIF. While the injection profile may follow a fixed wave-form specified by a simple injection protocol, e.g. a bolus or a 1 min infusion resulting from an automated syringe, there may be cases where a more complicated profile is introduced. In the latter case, the scanner FOV may allow the profile to be directly specified based on a suitable injection site ROI. The work here proposes that the statistical characteristics of a population of impulse response curves for a given tracer be used to formulate a suitable data-adaptive constrained regularisation method for estimation of the AIF. Combination of regularised AIF estimators extracted from the analysis of separate ROI data sets is also possible.

The outline of the paper is as follows: the basic theory and modelling approach is developed section 2. Illustrative examples involving brain imaging studies with PET are presented in section 3. Section 4 describes a series of numerical studies with different tracers that investigate the suitability of the impulse response model representation and the impact of the constraints used to ensure its identifiability when the data are impacted by dispersion and/or spillover from background. The paper concludes with discussion in section 5.

## 2. Theory

### 2.1. Impulse response representation of the AIF and modelling of blood ROI data

The AIF, denoted *C*_*p*_, is represented as a convolution between an unknown non-negative impulse response function, denoted ${ \mathcal R }$, and a known function, *C*_*I*_, specifying the profile of the injected tracer at the site of injection. The equation defining the AIF is

\begin{eqnarray*}{C}_{p}(t)={\int }_{0}^{t}{ \mathcal R }(t-s){C}_{I}(s){ds}\equiv { \mathcal R }\,\ast \,{C}_{I}[t].\end{eqnarray*}

Because *C*_*I*_ is known, the AIF is fully determined by the impulse response. Typically the injection profile is simply an indicator for the interval [0, *d*], where *d* > 0 is the duration of the injection indicated in the PET study protocol—see the illustrative examples in the next section. However, in situations where the injection site is in the scanner field of view, a direct measurement of *C*_*I*_, may also be possible.

The goal is to estimate the AIF, or equivalently the impulse response, based on information in a PET-measured time-course data for a ROI associated with a blood pool. The ROI data are assumed to be sampled at *n*-time points corresponding to mid-points (*t*_*i*_) of PET-scanning time-frames. Thus the ROI data are {*z*_*t*_, *t* = *t*_1_ < *t*_2_ < ,..., < *t*_*n*_} where ${z}_{{t}_{i}}$ is the measured PET activity in the *i*th time-frame of PET data acquisition. Of course, it is well-appreciated that the variability of a PET activity measurement over a given time-frame is dependent on the underlying true activity, the duration of the time-frame and on the decay-correction required to adjust for tracer half-life—see (Huesman 1984, Mou *et al* 2017), for example. Accordingly, it is assumed that there is sequence of known weights {*w*_*i*_, *i* = 1, 2,…,*n*} associated with the ROI data whose inverse values are approximately proportional to the variability of the individual time-frame activity measurements. Typically weights are proportional to the product of the duration of the data frame and the decay correction factor used to convert raw PET counts to activity units—see (Huesman 1984). Ideally, the blood region ROI time-course values would give a close approximation to the true AIF, however this may be questionable in cases where the blood region is impacted by either dispersion and/or background spillover effects. Dispersion effects are modelled by convolution of the AIF with a simple mono-exponential form—i.e. the dispersed AIF is ${E}_{\phi }\,\ast \,{C}_{p}[t]=\tfrac{1}{\phi }{\int }_{0}^{t}{e}^{-\tfrac{t-s}{\phi }}{C}_{p}(s){ds}$ with the parameter *ϕ* > 0 controlling the amount of dispersion involved. This approach to analysis of dispersion follows the well-established Kety–Schmidt model for a vascular flow network (Iida *et al* 1986). For spillover from background, it is assumed that there is a separate time-course {*S*_*t*_, *t* = *t*_1_ < *t*_2_ < ,..., < *t*_*n*_} describing the structure of the background pattern that might be impacting the ROI data. This background pattern might be derived from an ROI placed in tissue surrounding the blood region ROI. A less specific Patlak-style representation of background, see (Patlak *et al* 1983), may also be reasonable. This is defined as the cumulative integral of the injection profile, i.e. ${S}_{t}={\int }_{0}^{t}{C}_{I}(s-{\mathrm{\Delta }}){ds}$, where Δ is a suitable time-shift that aligns the Patlak-style representation with the measured ROI data. Given these representations, the blood region ROI data is modelled, up to an unknown error, *ϵ*, as a positive linear sum of contributions from the pure AIF, dispersion and spillover from background

\begin{eqnarray*}{z}_{{t}_{i}}={\alpha }_{1}{C}_{p}[{t}_{i}-{\mathrm{\Delta }}]+{\alpha }_{2}{E}_{\phi }\,\ast \,{C}_{p}[{t}_{i}-{\mathrm{\Delta }}]+{\alpha }_{3}{S}_{{t}_{i}}+{\epsilon }_{i}.\end{eqnarray*}

for *i* = 1, 2, …, *n*. The model errors are assumed to be independent random variables with mean zero and variance approximately inversely proportional to the set of weights, {*w*_*i*_, *i* = 1, 2, …, *n*}. The parameter Δ is an appropriate time-shift required to (temporally) align the AIF to the ROI data. The non-negative parameter vector ***α*** = (*α*_1_, *α*_2_, *α*_3_) controls the relative importance of the terms describing dispersion and spillover effects within the ROI. In the case that the background is represented as a convolution with the injection profile, i.e. ${S}_{t}=\tilde{S}\,\ast \,{C}_{I}(t-{\mathrm{\Delta }}]={\int }_{0}^{t}\tilde{S}(t-s){C}_{I}(s-{\mathrm{\Delta }}){ds}$ for a suitable $\tilde{S}$—the Patlak-style representation mentioned above is an example of this in which $\tilde{S}$ is constant -, the model can be expressed as

\begin{eqnarray*}\begin{array}{rcl}{z}_{{t}_{i}} &amp; = &amp; \{{\alpha }_{1}{ \mathcal R }+{\alpha }_{2}{E}_{\phi }\,\ast \,{ \mathcal R }+{\alpha }_{3}\tilde{S}\}\,\ast \,{C}_{I}[{t}_{i}-{\mathrm{\Delta }}]+{\epsilon }_{i}\\ &amp; \equiv &amp; \eta (\cdot | { \mathcal R },{\boldsymbol{\alpha }},\phi )\,\ast \,{C}_{I}[{t}_{i}-{\mathrm{\Delta }}]+{\epsilon }_{i}.\end{array}\end{eqnarray*}

### 2.2. Model identifiability

It is assumed that the unknown impulse response and the injection profile, *C*_*I*_, are both non-negative functions. In either equations (2) or (3), there is obvious ambiguity between the scales of the *α*_1_ and *α*_2_ and the scale of ${ \mathcal R }$. Somewhat arbitrarily, it is assumed that IR is normalised so that ${\int }_{0}^{\infty }{ \mathcal R }(t){dt}=1$. For technically reasons, it is simpler to discuss the identifiability issue when ${ \mathcal R }$ is normalised. The alternative to normalising ${ \mathcal R }$ is to set *α*_1_ = 1 and allow ${ \mathcal R }$ to be free. The numerical implementation uses the latter approach. Note normalisation impacts the scale of the AIF in equation (1). However, since it is expected that at least one of *α*_2_ (dispersion) and/or *α*_3_ (spillover) are non-zero, some supplementary information beyond the blood region ROI data is needed to correctly scale the AIF. In this context some possible methods for scaling the estimated AIF are discussed in section 2.4 below. With ${ \mathcal R }$ normalised, it is still not certain that it can be determined uniquely from the models specified in equations (2) or (3). Indeed even with idealised continuously sampled, noise-free data, the presence of dispersive or spillover components, makes it impossible to uniquely determine the normalised ${ \mathcal R }$. The inherent non-identifiability associated with dispersion may be overcome if it is assumed that an integral value, ${\mu }_{1}={\int }_{0}^{\infty }t{ \mathcal R }(t){dt}$, is known. Non-identifiability associated with spillover can be addressed whenever a suitable a contrast ratio value, ${\mu }_{2}={ \mathcal R }({T}_{b})/{ \mathcal R }({T}_{a})$, for the impulse response at distinct time points *T*_*a*_ and *T*_*b*_, is known. A formal identifiability result and its proof are provided in appendix A. In view of the result, if the ROI data are suspected of being influenced by dispersion or spillover from background then the scheme for estimation of ${ \mathcal R }$ based on equation (3) needs to incorporate suitable constraints, such as fixed values for *μ*_1_ and *μ*_2_ above. This is developed next. If there are previously gathered data sets of directly sampled arterial blood curves, these can be used to determine suitable values for *μ*_1_ and *μ*_2_ for specifying the constraints to apply in cases where dispersion or spillover might be a concern—see section 4.

### 2.3. Estimation with and without constraints

For estimation, the impulse response, ${ \mathcal R }$, is represented as a positive linear combination of a set of *K* basis elements {*B*_*k*_ , *k* = 1, 2, ...,* K*}. These elements are piecewise linear forms defined in terms of a set of fixed tracer-specific knots {0 = *t*_0_ < *t*_1_ < ... < *t*_*K*_}. The last knot, *t*_*K*_, is selected to be beyond the temporal duration of the PET study.

\begin{eqnarray*}{B}_{k}(t)=\left\{\begin{array}{ll}1 &amp; \,,\,\,\,0\lt t\lt {t}_{k}\\ 1-\frac{t-{t}_{k-1}}{{t}_{k}-{t}_{k-1}} &amp; \,,\,\,\,{t}_{k-1}\leqslant t\lt {t}_{k}\\ 0 &amp; \,,\,\,\,t\gt {t}_{k}\end{array}\right..\end{eqnarray*}

**Figure 1.**
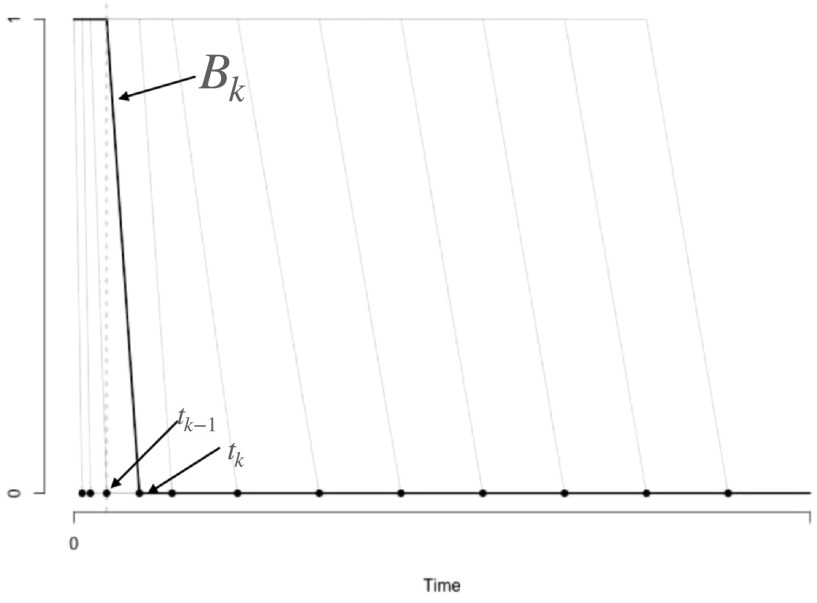
Sample basis elements {*B*_*k*_, *k* = 1, 2, ..., *K*} used for representation of the Impulse Response - grey lines. *B*_*k*_ (black line) is defined in terms of *t*_*k*−1_ and *t*_*k*_—see equation (4). Associated knot points {*t*_*k*_, *k* = 1, 2, ...,* K*} are shown with solid dots.

Figure 1 shows a typical configuration of basis elements. Note that while smoother basis elements could be considered, the results in sections 3 and 4 will show that the piecewise linear form appears to provide reasonable approximations to the target AIFs in the examples considered. The linear form do simplify the evaluation of convolutions with the injection wave-form.

${ \mathcal R }(t| {\boldsymbol{\theta }})={\sum }_{k\,=\,1}^{K}{\theta }_{k}{B}_{k}(t)$, with ***θ*** = (*θ*_1_, ...*θ*_*K*_), the unknown parameter-vector. With ***θ*** is non-negative, ${ \mathcal R }$ is non-negative and monotone decreasing. As mentioned earlier, the numerical implementation fixes *α*_1_ = 1 and ${ \mathcal R }$ is not normalised. Integral and contrast ratio constraints corresponding to *μ*_1_ and *μ*_2_ are implemented as linear constraints on the ***θ*** coefficients

\begin{eqnarray*}\begin{array}{rcl}{\mu }_{1} &amp; = &amp; \displaystyle \frac{{\int }_{t}t{ \mathcal R }(t| {\boldsymbol{\theta }}){dt}}{{\int }_{t}{ \mathcal R }(t| {\boldsymbol{\theta }}){dt}}\Longleftrightarrow \displaystyle \sum _{k}{A}_{k1}{\theta }_{k}\equiv \displaystyle \sum _{k}{\int }_{t}({\mu }_{1}-t){B}_{k}(t){dt}\,{\theta }_{k}=0\\ {\mu }_{2} &amp; = &amp; \displaystyle \frac{{ \mathcal R }({T}_{b}| {\boldsymbol{\theta }})}{{ \mathcal R }({T}_{a}| {\boldsymbol{\theta }})}\Longleftrightarrow \displaystyle \sum _{k}{A}_{k2}{\theta }_{k}\equiv \displaystyle \sum _{k}({\mu }_{2}{B}_{k}({T}_{a})-{B}_{k}({T}_{b}))\,{\theta }_{k}=0\end{array}\end{eqnarray*}

**A** = [*A*_·1_, *A*_·2_]. Let *BI*_*k*_ = *B*_*k*_ ∗ *C*_*I*_ for *k* = 1, 2,...,*K*. With $\pi =\tfrac{{\alpha }_{2}}{{\alpha }_{1}+{\alpha }_{2}}$, *ϕ* and Δ fixed, let *X*_*ik*_[*π*, *ϕ*, Δ] = (1 − *π*)*BI*_*k*_(*t*_*i*_ − Δ) + *π* *E*_*ϕ*_ ∗ *BI*_*k*_(*t*_*i*_ − Δ) for *k* = 1, 2, ...*K* and *i* = 1, 2, ...,* n*. Also let ${X}_{i(K+1)}[\pi ,\phi ,{\mathrm{\Delta }}]={S}_{{t}_{i}}$. Augment ***θ*** to include a component corresponding to *α*_3_ and augment the constraint matrix **A** with a final row of zeros. Model fit is evaluated by the weighted sum of squared deviations between the ROI data values, ${z}_{{t}_{i}}$, and the corresponding model predictions, ${X}_{i}[\pi ,\phi ,{\mathrm{\Delta }}]^{\prime} {\boldsymbol{\theta }}$. The weights are {*w*_*i*_, *i* = 1, 2, ..., *n*}—discussed following equation (2). The optimal parameters minimise

\begin{eqnarray*}\begin{array}{l}WRSS({\boldsymbol{\theta }},\pi ,\phi ,{\mathrm{\Delta }})=\displaystyle \sum _{i=1}^{n}{w}_{i}{\left[{z}_{{t}_{i}}-{X}_{i}[\pi ,\phi ,{\mathrm{\Delta }}]^{\prime} {\boldsymbol{\theta }}\right]}^{2}\\ \quad \mathrm{subject}\,\mathrm{to:}{\boldsymbol{\theta }}\geqslant 0\,,\,{\bf{A}}^{\prime} {\boldsymbol{\theta }}={\bf{0}}.\end{array}\end{eqnarray*}

There are three intrinsically non-linear parameters—(*π*, *ϕ*, Δ). For fixed, (*π*, *ϕ*, Δ), the optimal ***θ*** is found by application of quadratic programming. The procedure of (Goldfarb and Idnani 1983) implemented in the *quadprog* package in (R Core Team 2021) is used. This gives the reduced weighted residual sum of squares objective function

\begin{eqnarray*}WRSS(\pi ,\phi ,{\mathrm{\Delta }})={\min }_{\{{\boldsymbol{\theta }}\geqslant 0\,,\,{\bf{A}}^{\prime} {\boldsymbol{\theta }}={\bf{0}}\}}[WRSS({\boldsymbol{\theta }},\pi ,\phi ,{\mathrm{\Delta }})].\end{eqnarray*}

The gradient based optimisation algorithm of (Byrd *et al* 1995) and implemented in (R Core Team 2021) by the function *optim*, is used to evaluate the values of (*π*, *ϕ*, Δ) that minimise the reduced *WRSS* function in equation (7). The optimised ***θ*** is denoted $\hat{{\boldsymbol{\theta }}};$ the corresponding optimised values for (*π*, *ϕ*, Δ), is $(\hat{\pi },\hat{\phi },\hat{{\mathrm{\Delta }}})$.

It is important to demonstrate that the imposition of constraints does not substantially impact the ability to approximate the true (unknown) AIF using a constrained IRF. Studies in section 4.1 consider this issue by examining a collection of data where the true AIF is available and it is possible to consider how well the AIF can be represented using the IRF representation in equation (1), with or without the constraints in equation (5) that give the potential to recover the AIF from data that might involve dispersion or spillover contamination. In the context of the studies in 4.1, dispersion (*π* = 0) and spillover (*α*_3_ = 0) are fixed and the reduced *WRSS* function is just optimised over Δ. Direct arterially sampled data are first analyzed without linear constraints relating to *μ*_1_ and *μ*_2_. This done by removing the constraints associated with the matrix **A** above. Results of this analysis across, a series of PET studies with a given tracer, is used to specify suitable values of (*μ*_1_, *μ*_2_) in equation (5). Directly sampled arterially data also examined with the (*μ*_1_, *μ*_2_) constraints imposed. Comparison between the results of the constrained and unconstrained analysis provides a quantitative assessment of the impact of the constraints needs for practical image-based extraction of AIFs with that tracer.

### 2.4. Empirical regularisation

The analysis of a historical collection of, say *K*, directly sampled AIFs, provides insight into the statistical behaviour of the resulting impulse response parameters $\{{\hat{{\boldsymbol{\theta }}}}_{k},k=1,2,\,...,\,K\}$. The *a-priori* data, $\{{\hat{{\boldsymbol{\theta }}}}_{k},k=1,2,...,K\}$, can be used to augment the weighed least squares in (6) in order to create a potentially more reliable regularised criterion for estimation of the impulse response based on image-extracted ROI data. The use of regularisation of this type in function estimation is discussed in the classic work (Wahba 1990). There are well-established connections between regularisation, shrinkage and more sophisticated Bayesian estimation procedures—see (Wahba 1990) for a discussion. In the present setting, the *a-priori* data leads to the specification of a regularised objective function as a linear combination of data fit, measured by the weighted residual sum of squares, and Mahalanobis deviation between ***θ*** and the mean of the *a priori* data.

\begin{eqnarray*}{\ell }_{\lambda }({\boldsymbol{\theta }},\pi ,\phi ,{\mathrm{\Delta }})={WRSS}({\boldsymbol{\theta }},\pi ,\phi ,{\mathrm{\Delta }})+\lambda ({\boldsymbol{\theta }}-{{\boldsymbol{\mu }}}_{\theta })^{\prime} {{\boldsymbol{\Sigma }}}_{\theta }^{-1}({\boldsymbol{\theta }}-{{\boldsymbol{\mu }}}_{\theta }),\end{eqnarray*}

where *λ* > 0. Here ***μ***_*θ*_ and **Σ**_*θ*_ are the mean and covariance of the data $\{{\hat{{\boldsymbol{\theta }}}}_{k},k=1,2,...,K\}$. Note with *λ* = 0, the minimizer of (8) reduces to the solution of (6); while as *λ* → ∞ , the optimal ***θ*** reduces to the *a priori* mean—***μ***_*θ*_. For fixed choice of *λ*, the minimisation of *ℓ*_*λ*_(***θ***, *π*, *ϕ*, Δ) in (8), subject to ***θ*** = 0 and ${\bf{A}}^{\prime} {\boldsymbol{\theta }}={\bf{0}}$, follows the same approach as that used for minimisation of (6). A generalised cross-validation criterion is used for selection of *λ*—see (O’Sullivan and Wahba 1985, Wahba 1990).

### 2.5. Pooling results form separate ROIs

In an imaging setting it may happen that there are several, *e*.g. *J* > 1, potential ROI time-courses available as possible inputs for AIF extraction. In such a situation the combination or pooling of the AIF extractions corresponding to each ROI, $\{{\hat{{\boldsymbol{\theta }}}}_{1},{\hat{{\boldsymbol{\theta }}}}_{2},...,{\hat{{\boldsymbol{\theta }}}}_{J}\}$, may be of interest. Based on the analysis provided in (Gu *et al* 2021) the optimal statistical combination of separate estimators is the covariance-weighted average of the individual estimators, i.e.

\begin{eqnarray*}\tilde{{\boldsymbol{\theta }}}={\left[{\hat{{\boldsymbol{\Sigma }}}}_{1}^{-1}+...+{\hat{{\boldsymbol{\Sigma }}}}_{J}^{-1}\right]}^{-1}({\hat{{\boldsymbol{\Sigma }}}}_{1}^{-1}{\hat{{\boldsymbol{\theta }}}}_{1}+...+{\hat{{\boldsymbol{\Sigma }}}}_{J}^{-1}{\hat{{\boldsymbol{\theta }}}}_{J}),\end{eqnarray*}

where ${\hat{{\boldsymbol{\Sigma }}}}_{j}$ is the covariance of the *j*th estimator—this might evaluated using a suitable bootstrapping process (O’Sullivan *et al* 2021). The current implementation uses a more simplified weighted averaging process. For each estimator, the proportion of the total spillover-corrected time course variance explained by the fitted AIF is evaluated. For a generic ROI, this measure is given by

\begin{eqnarray*}{\hat{\sigma }}^{2}=\displaystyle \frac{{\sum }_{i}{w}_{i}{\left[{z}_{i}-{X}_{i}[\hat{\pi },\hat{\phi },\hat{{\mathrm{\Delta }}}]^{\prime} \hat{{\boldsymbol{\theta }}}\right]}^{2}}{{\sum }_{i}{w}_{i}{\left[{z}_{i}-{\hat{\alpha }}_{3}{S}_{i}\right]}^{2}}\end{eqnarray*}

Evaluating these for each ROI, gives $\{{\hat{\sigma }}_{1}^{2},...,{\hat{\sigma }}_{J}^{2}\}$ and assuming ${\hat{{\boldsymbol{\Sigma }}}}_{j}\approx {\hat{\sigma }}_{j}^{2}{\mathrm{\Sigma }}$, the pooled estimator reduces to a simple weighted average

\begin{eqnarray*}\bar{{\boldsymbol{\theta }}}=\displaystyle \sum _{j}{\hat{\omega }}_{j}{\hat{{\boldsymbol{\theta }}}}_{j}\equiv \frac{{\hat{\sigma }}_{1}^{-2}{\hat{{\boldsymbol{\theta }}}}_{1}+...+{\hat{\sigma }}_{J}^{-2}{\hat{{\boldsymbol{\theta }}}}_{J}}{{\hat{\sigma }}_{1}^{-2}+...+{\hat{\sigma }}_{J}^{-2}}.\end{eqnarray*}

This is the approach used in the illustrations presented in section 3. In those cases AIF extraction results from a set of *J* = 40 segmentation-derived ROIs are combined. Remarks: it should be appreciated that the parameters (*π*, *ϕ*, Δ) that arise for individual ROIs carry no specific information about the impulse parameters, ***θ***, or the AIF. These parameters are quite important however as they allow the possibility to combine information about the AIF from areas with much different kinetic patterns as reflected by their unique spillover and dispersion effects. In a statistical estimation context, the parameters (*π*, *ϕ*, Δ) would be best viewed as nuisance parameters. Direct averaging of separate ROIs would typically create an ROI with complex kinetics which would not be accessible to an analysis in terms of the basic model form in equations (2) and (3).

### 2.6. AIF scaling

The estimated AIF corresponding to the estimated impulse response $\hat{{ \mathcal R }}$ is $\hat{{ \mathcal R }}\,\ast \,{C}_{I}$. This AIF value is denoted ${\bar{C}}_{p}$. As discussed in section 2.2 above, when there is dispersion or spillover, the estimated AIF needs to be scaled in order to reflect the correct units of PET activity—e.g. KBq or mCi per ml of blood. Three potential ways to scale the ${\bar{C}}_{p}$ to get the correctly scaled value, denoted ${\hat{C}}_{p}$, are:

(i) Blood Sample: If an arterial blood measurement is available at time *t*_*B*_, scale the AIF so that
  \begin{eqnarray*}{\hat{C}}_{p}({t}_{B})={d}_{B},\end{eqnarray*}
  where *d*_*B*_ is the direct arterial blood measurement. Of course it is assumed that this measurement is reliable—i.e. substantially unbiased with little noise. Note the measurement could be either an arterial or a venous sample taken at a time when the arterial and venous blood can be assumed to have equilibrated. Arterial—venous equilibration times are typically established as part of basic dosimetry studies for the tracer. In the illustrations presented in section 3, blood scaling is applied using at a time-point where arterial and venous signals would be expected to have equilibrated. As ${\hat{C}}_{p}$ is the tracer molecule activity, in cases where a significant part of circulating activity relates to metabolites of the injected tracer, the raw blood activity may need some analysis to determine the activity associated with circulating tracer molecules. This is a standard process needed for some PET tracers—e.g. (Spence *et al* 2003, Muzi *et al* 2006, 2009). One technical point with this approach is that care must be taken to match blood timing with the timing of PET acquisition.
(ii) Physiologic: if there is an obvious well resolved ROI in the FOV with known physiology then the estimated AIF might be scaled so that the computed physiologic parameter for the ROI matches physiologic expectation. For example for a blood-rich ROI such as the ascending/descending aorta, the ROI might be expected to behave as a vascular network with a single source and sink but no extraction of tracer within the network—see (Meier and Zierler 1954). As a result, if the time-course for the ROI were analyzed using the correctly scaled AIF to derived an estimated tissue residue, i.e. ${z}_{t}\approx \hat{R}\,\ast \,{\hat{C}}_{p}[t-\hat{{\mathrm{\Delta }}}]$, one would expect that the integrated residue, ${\hat{R}}_{\bullet }={\int }_{0}^{{T}_{E}}\hat{R}(t){dt}$, or total tissue volume to be unity, see (Meier and Zierler 1954, Gu *et al* 2021). Thus if the ROI data were analyzed using the AIF, ${\bar{C}}_{p}$, i.e. $\hat{R}\,\ast \,{\hat{C}}_{p}[t-\hat{{\mathrm{\Delta }}}]=\tilde{R}\,\ast \,{\bar{C}}_{p}[t-\hat{{\mathrm{\Delta }}}]$, the integral of the tissue residue $\tilde{R}$ over the duration of the study could be used to scale
  \begin{eqnarray*}{\hat{C}}_{p}=\displaystyle \frac{{\tilde{R}}_{\bullet }}{{\hat{R}}_{\bullet }}\times {\bar{C}}_{p}\,\,{\mathrm{where}}\,\,{\tilde{R}}_{\bullet }={\int }_{0}^{{T}_{E}}\tilde{R}(t){dt}\end{eqnarray*}
  Note a net retention of tracer in the ROI at the end of the study (${\tilde{R}}_{\bullet }$ underestimated) or a significantly reduced recovery coefficient for the ROI (${\hat{R}}_{\bullet }\lt 1$) would lead to an under-estimation of the true AIF scale.
(iii) Injected dose per unit blood volume of subject: Historical collections of directly sampled arterial blood data provide an opportunity to empirically evaluate the relation between the activity in the arterial blood at a fixed (late) time *T*_*E*_ and the injected activity (*V*_*i*_ *τ*—product of volume of injection and activity per unit volume injected) per unit blood volume of the subject, BV, assessed using Nadler’s formula (Nadler *et al* 1962). Thus there would be an empirical basis to predict the value of the arterial activity based on the injected activity and the subject’s BV value. This would allow the AIF to be scaled so that ${\hat{C}}_{p}({T}_{E})$ matches the value predicted from analysis of the historical data.

\begin{eqnarray*}{\hat{C}}_{p}({T}_{E})=\hat{\beta }\tau \frac{{V}_{I}}{{BV}},\end{eqnarray*}

where *BV* is the subject’s Nadler-computed total blood volume, *τ* is the injected activity per ml and *V*_*I*_ is the volume of the injection. $\hat{\beta }$ would be determined from the analysis of historical data—see (Huang and O’Sullivan 2014, Xiu *et al* 2023) for exploration of this approach.

## 3. Illustrations

### 3.1. Data sets

We focus on a set of four PET brain imaging studies conducted as part of NIH-funded research studies at University of Washington Medical Center. Two of the four studies used [^18^F]-labeled fluorodeoxyglucose (FDG) and [^15^O]-labeled water (H2O) studies in normal subjects; the other two studies were with [^18^F]-labeled fluoro-thymidine (FLT) and [^11^C]-labeled carbon dioxide (CO2) in post-surgery brain tumor patients. Full details of the individual studies are given in the reports (Spence *et al* 1998, Mankoff *et al* 2003, O’Sullivan *et al* 2014). All scanning was carried out on a 35-plane GE-Advance tomograph with a 15cm axial field of view and a within-plane resolution of 2.25 mm. Raw data were acquired in 2D mode and reconstructed using a traditional filtered-backprojection algorithm. The injected doses in the studies were as follows: 379 MBq of [^18^F]-labeled fluorodeoxyglucose, 144 MBq of [^18^F]-labeled fluorothymidine, 349 MBq of [^11^C]-labeled carbon dioxide and 457 MBq of [^15^O]-labeled water. In the case of FDG, FLT, and CO2, the dose was mixed in a 7–10 ml volume and injected over 1 min using a constant infusion pump; for H2O the dose was mixed in a 4 ml volume and injected as a bolus (5 second duration). The injection profiles, *C*_*I*_ in equation (1), corresponding to these were squares with duration (d) of 1 min for FDG, FLT, and CO2 and of duration 5 s for H20. FDG dynamic imaging was carried out over 90 minutes according to the following acquisition sequence (number of frames and their durations given): 1(1 min) pre-injection, 4 (15 s), 4 (30 s), 4(1 min), 4 (3 min) and 14 (5 min); FLT scanning was done over 90 min according to: 10 (10 s), 4 (20 s), 4 (40 sec), 5(2 min), 4 (3 min) and 13 (5 min); CO2 scanning was done over 60 min according to: 4 (20 s), 4 (40 s), 4 (40 s), 4(1 min), 4 (3 min) and 8 (5 min); finally, dynamic H20 imaging was carried out over 8.25 minutes as : 1(1 min) pre-injection, 5 (3 s), 10 (6 s), 12 (10 s), 8 (15 s) and 6 (20 s). Each study also had a directly measured blood time-course, obtained by catheterised arterial sampling. We use this for scaling, e.g. equation (12). The sampled data makes it possible to compare our extracted AIF with the true arterial time-course.

### 3.2. AIF extractions

The scanner resolution and physiology makes identification of arterial blood-pools impractical. In light of this a segmentation procedure from (O’Sullivan *et al* 2014, Gu *et al* 2021) is applied to recover a collection of 40 time-courses from the raw dynamic data. Each of the 40 segments is used as an ROI for AIR extraction. Results from these extractions are combined using the method in section 2.5. Segment time-courses are first analysed using non-parametric residue analysis procedure based on the population average IR (***μ***_*θ*_)—(Gu *et al* 2021). The suitably shifted integrated AIF corresponding to ***μ***_*θ*_ is then taken to represent the spillover pattern for the segment ROI time-course. The regularised IR extraction process is applied to each segment time-course and the averaging process in (11) used to evaluate the overall extracted IR and the associated normalised AIF. The estimated normalised AIFs is scaled using a single blood sample - equation (12). The full analysis was implemented in R (R Core Team 2021).

**Figure 2.**
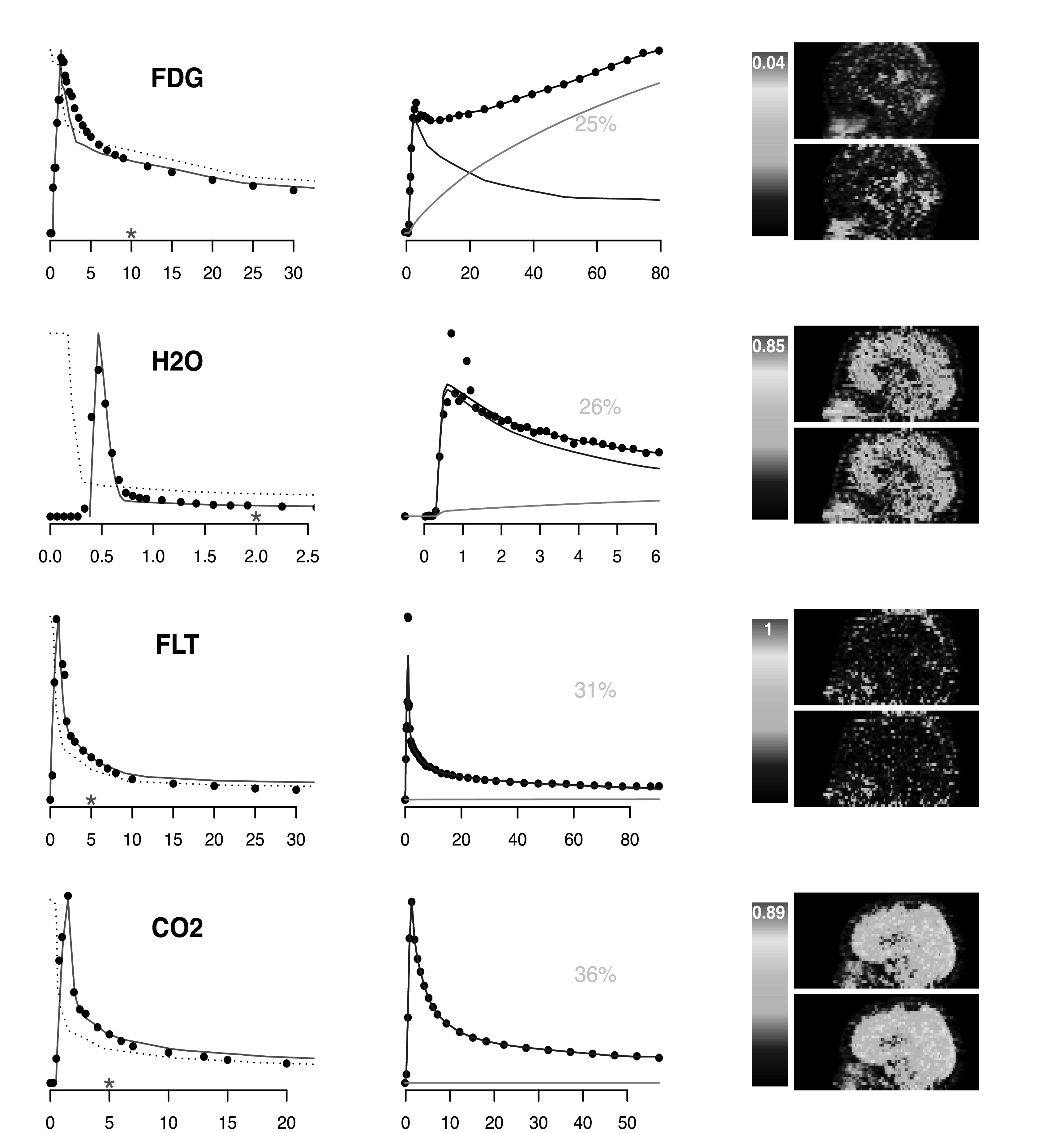
Sample AIF extractions for a set of Cerebral PET imaging studies in different subjects and with different tracers (FDG, FLT, CO2 and H2O). Left: recovered AIF (red) and the sample arterial data (black dots). Red asterisk is time-point (*t*_*B*_ in (12)) The dashed curve (not on the same scale) is the estimated impulse response ($\hat{{ \mathcal R }}$ ). Middle: analysis of the segment time-course (black dots) given the highest weight (weight is grey, see equation (11)) for AIF extraction. The fitted model is shown by the solid black line. Estimated spillover and dispersion components are in green and blue, respectively—see equation (2). Right: Volume (mg ml^–1^) of tracer atoms with estimated transit times less than 15 s. Results are for selected sagittal slices using the directly sampled AIF (top) and the image extracted AIF (bottom).

The extracted AIFs are shown in figure 2. Note that although a set of 40 segments are derived for each dataset, blood extractions are substantially based on 4–8 segment time-courses with the greatest weight. Figure 2 presents the analysis of the ROI data for the segment given the greatest weight, via equation (11) in the AIF extraction process. Note that for FDG and H2O the ROI with the highest weight shows significant dispersion and background spillover. With FLT and CO2, the most highly weighted segment ROI shows significant dispersion but the impact of background is much less pronounced. There is good agreement between the extracted and the true, directly sampled, AIFs. The extraction AIFs are quite close to the correct arterially sampled ones. The FDG case is most divergent but it still performs remarkably well. The physiologic approach in equation (13) was also applied for scaling. Physiologically scaled AIFs are lower than the direct sample values: 11% for H20; 25% for FDG, 29% for CO2 and 68% for FLT. These deviations might be partially explained by the scanner resolution so a suitable adjustment for the recovery coefficient of the blood ROI may help to reduce the error in physiologic scaling. This merits more detailed investigation. The central column in figure 2 shows the fit of the most heavily weighted ROI data set involved in the AIF extraction process—the weight is provided in the figure - see equation (11). This is an empirical demonstration of the conformity of the ROI data to the model in equation (3). Given that (3) is used as a basis for the AIF extraction, it is important see if the modelling is reasonable. The dispersion and spillover patterns for each ROI are also shown. In the case of FDG, there is a substantial spillover pattern largely following the shape of the integrated AIF. This is the familiar FDG uptake characteristic associated with white and grey matter in the brain—see (Patlak *et al* 1983).

Using the NPRM technique (O’Sullivan *et al* 2014, Gu *et al* 2021), estimates of voxel-level residues were computed used to evaluate the volume of tracer atoms with transit times less than 15 s. Sagittal slices of this information for directly sampled and image extracted AIFs are shown in figure 2. The images are quite noisy—perhaps not surprising given the sampling—but the general patterns are the same for both the true and extracted AIFs. There are sharp differences between the general pattern of rapid transit through tissue for the the freely diffusible H2O and CO2 molecules and the much more restricted patterns for FDG and especially FLT (which does not cross the blood-brain barrier). There is a marked difference between FDG and the other tracers, all of which show regions where the volumes of rapidly progressing tracer molecules exceed 0.85 ml mg^–1^. With FDG the highest such volumes are around 0.04 ml mg^–1^ but for most brain regions the typical volume is closer to 0.01 ml mg^–1^. For FDG and FLT, the higher volumes of the rapidly transiting tracer atoms are seen to be associated with the nasal cavity and major blood vessels such as the internal carotids and sagittal sinus. A full set of kinetic information can be derived using the NPRM technique. The volumes images presented are those associated with tracer molecules that remain in tissue for a short period of time (15 s is not a long time in the context of these studies); these images were selected as they would given an indication of more vascular aspects of the tracers distribution within the tissue. The primary purpose of images is to highlight that the information related to a highly vascular tissue characteristic derived for the directly sampled AIF is substantially similar to that derived from the image-extracted AIF. A detailed investigation of the AIF extraction scheme and its impact on the recovery of the full range of kinetics would of course be worthwhile. This is a topic for future work.

## 4. Numerical studies and results

Two sets of studies are described here. In section 4.1 it is shown that the impulse response representation of the AIF is quite adequate and also that there is negligible loss in the accuracy of the representation by imposing the constraints in equation (5) on the impulse response. Section 4.2 uses simulation to examine the reliability of the AIF extraction process in a setting where the input data is impacted by dispersion and spillover effects. A database of directly sampled arterial blood curves (AIFs) were used in these studies. The collection of AIFs used arise from PET studies conducted over a 25 year period at the University of Washington Medical Center. These data were provided through a collaborative agreement with a number of NIH-supported principal investigators at the University of Washington in the context of a research project at University College Cork. Arterial blood sampling used a system reported in (Graham and Lewellen 1993). The AIF data here for the FDG, FLT, H2O and CO2 tracers described in the illustration^1^ 
^1^
None of the analyses here included AIF data from the cases used for the illustration in section 3.—see figure 2 - and for another four ^11^C-labeled tracers of glucose (GLC), verapamil (Verap), thymidine (TdR) and acetate (ACT). While H20 was injected as a bolus, other studies involved 1-minute infusion of tracer. Detailed protocols for individual tracers are described in (Spence *et al* 1998) for FDG and GLC; (Muzi *et al* 2006) for FLT, (Spence *et al* 2003) for ACT, (Wells *et al* 2002) TdR and CO2, and (Muzi *et al* 2009) for Verap and H2O. The numbers of available AIFs varied by tracer—see table 1.

**Table 1.** Tracer impulse response characteristics derived from analysis of sampled Arterial data. Estimated percentiles (25%, 50% and 95%) of tracer molecules remaining in circulation for at least 5 s after introduction. Note tracers are ordered according to the 50’th percentile (half-life) value. Values given are summaries - mean ± standard errors of mean - recovered from individual impulse responses. *N* is the number of AIFs for each tracer.

PET Tracer	Aconym	N	25% [sec]	50% [sec]	95% [min]
^15^O-H_2_O	H2O	38	7.4 ± 0.1	9.5 ± 0.2	1.1 ± 0.1
^11^C-Verapamil	Verap	26	23.2 ± 2.2	29.0 ± 2.5	2.9 ± 0.4
^18^F-Fluorothymidine	FLT	33	24.5 ± 1.8	30.6 ± 1.8	8.9 ± 0.6
^11^C-Acetate	ACT	10	25.3 ± 6.9	37.9 ± 7.2	8.6 ± 0.6
^11^C Thymidine	TdR	10	28.6 ± 3.7	39.4 ± 3.9	7.3 ± 1.8
^11^C-CO_2_	CO2	9	38.7 ± 3.0	44.9 ± 3.1	15.4 ± 1.3
^11^C-Glucose	GLC	48	67.7 ± 5.7	75.0 ± 5.3	54.0 ± 1.7
^18^F-Fluorodeoxyglucose	FDG	48	72.8 ± 5.5	78.9 ± 4.4	47.6 ± 1.4

### 4.1. Impulse response modelling approach with and without constraints

**Figure 3.**
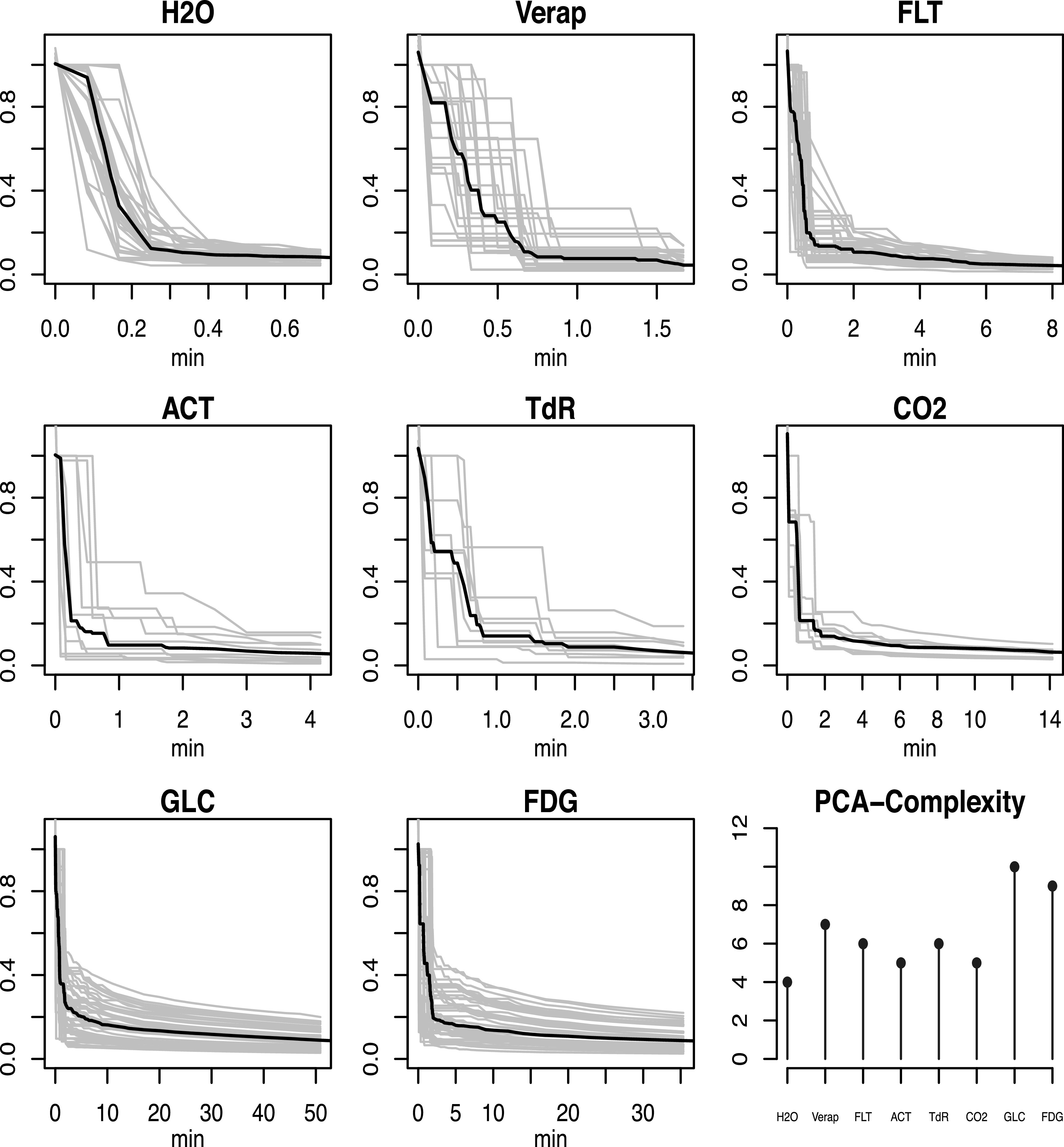
Estimated impulse response functions (grey lines) based on analysis of the directly sampled AIF data for each tracer. The median impulse response for the tracer is shown in black. The temporal range of plots stops at a point that at 10% of the peak for the mean impulse response. Bottom right plot shows the number of principal components needed to account for 90% of the correlation matrix of the estimated set of impulse response curves.

The ability of the impulse response modelling approach, with and without the constraints needed to ensure identifiability in the context of spillover of dispersion, was explored using the directly sampled AIF data. Figure 3 shows the estimated impulse responses, normalised to the value at 4 s, for all AIFs examined. The impulse responses for H2O shows the most precipitous temporal decline; FDG is the most persistent. Table 1 summarises the averages and standard errors of selected percentiles of the normalised impulse response for each tracer.

The 50’th percentile of the normalised impulse response can be the interpreted as the half-life for the circulation of tracer molecules in the arterial system. Apart from H2O which has a short 10 s half-life, tracer molecule half-lives are between 30 and 80 s. Figure 3 also evaluates the number of eigenvectors of the impulse response correlation matrix needed to explain 90% of the sum of its trace. In standard multivariate analysis the eigenvectors of the correlation define the principal components of variation in the standardized data. In the present setting the number of components needed to explain 90% of the total variability gives a sense of the effective dimensionality of the collection of impulse responses—see (Mardia *et al* 1979). This gives insight into how the penalty term in the regularised extraction criterion in equation (8) restricts the parameter space (Wahba 1990). This information gives a sense of the complexity or effective number of free parameters associated with estimating the impulse response from measured data—direct or recovered from PET scans.

**Figure 4.**
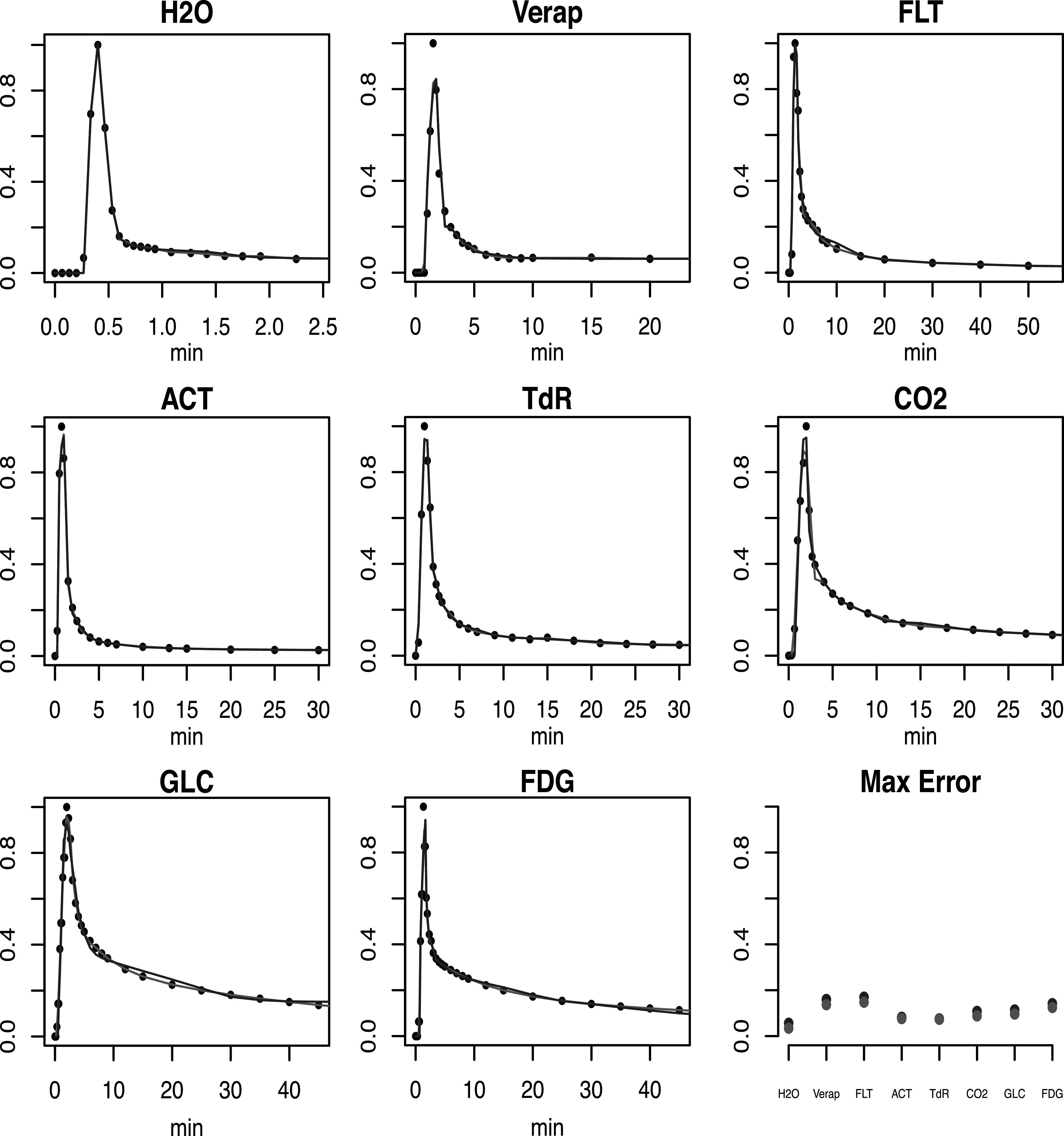
Sample unconstrained (red) and constrained (blue) fits to a particular AIF case for each tracer. The case chosen corresponds to the upper quartile (75th percentile) of the residual RMS errors for the unconstrained model fits to the set of tracer AIFs. Black dots are the raw data. Bottom right plot shows an average, over the tracer AIFs, of the maximum absolute error for the unconstrained (red) and constrained (blue) models.

Figure 4 shows representative results for a particular AIF within the series available for each tracer. The selected dataset is the case corresponding to the 75th percentile value for the residual sums of squares mis-fits of the constrained model. Thus for each tracer 75% of the AIF curves will look better than what is shown in figure 4. The model fits are seen to be very good both, with or without constraints. While careful analysis shows significant percent increases in the residual RMS error when using the constrained model, this mostly arises because the unconstrained model has almost no error, except perhaps in the area of the peak. The average maximum absolute error rate at the peak for the constrained and unconstrained model fits are very similar—see figure 4. It may be concluded that in practical terms constraining the impulse response, does not impact the representation of the AIF.

### 4.2. Estimation of the AIF from simulated PET data

Here we examine performance in a setting where sampling characteristics matched to the PET studies in section 3. For these studies, the ROI time-course data are simulated with Poisson-like variability. The true decay-corrected concentration, *C*_*T*_, for the ROI is assumed to be a weighted sum of the true AIF (*C*_*p*_), an dispersed signal (*C*_*d*_) obtain by convolution with an exponential with half-life matched to the data in table 1 and a spillover signal proportional to the cumulative integral of the AIF

\begin{eqnarray*}{C}_{T}(t)=(1-{p}_{d}){C}_{p}(t)+{p}_{d}{C}_{d}(t)+{p}_{b}\bar{S}(t),\end{eqnarray*}

where 0 < *p*_*d*_, *p*_*b*_ < 1 with $\bar{S}$ scaled so that its value at the end of the study is unity. The parameters *p*_*d*_ and *p*_*b*_ control the dispersive and spillover effects. A number of cases were considered, ranging from situations where the data is substantially free of contamination, (*p*_*d*_, *p*_*b*_) = (0.1, . 125), to situations where the contamination is quite significant (*p*_*d*_, *p*_*b*_) = (0.6, 2.0).

Decay-corrected PET ROI data {*z*_*i*_, *i* = 1, 2,...,*n*} were generated to have mean *C*_*T*_(*t*_*i*_), here *t*_*i*_ is at the mid-point of the *i* data-frame, and variance ${\sigma }_{i}^{2}$ proportional to the mean appropriately adjusted for decay (${e}^{\tau {t}_{i}}$) and frame duration (Δ_*i*_) —i.e. ${\sigma }_{i}^{2}\propto {C}_{T}({t}_{i})\tfrac{{e}^{\tau {t}_{i}}}{{{\mathrm{\Delta }}}_{i}}$ i.e.

**Figure 5.**
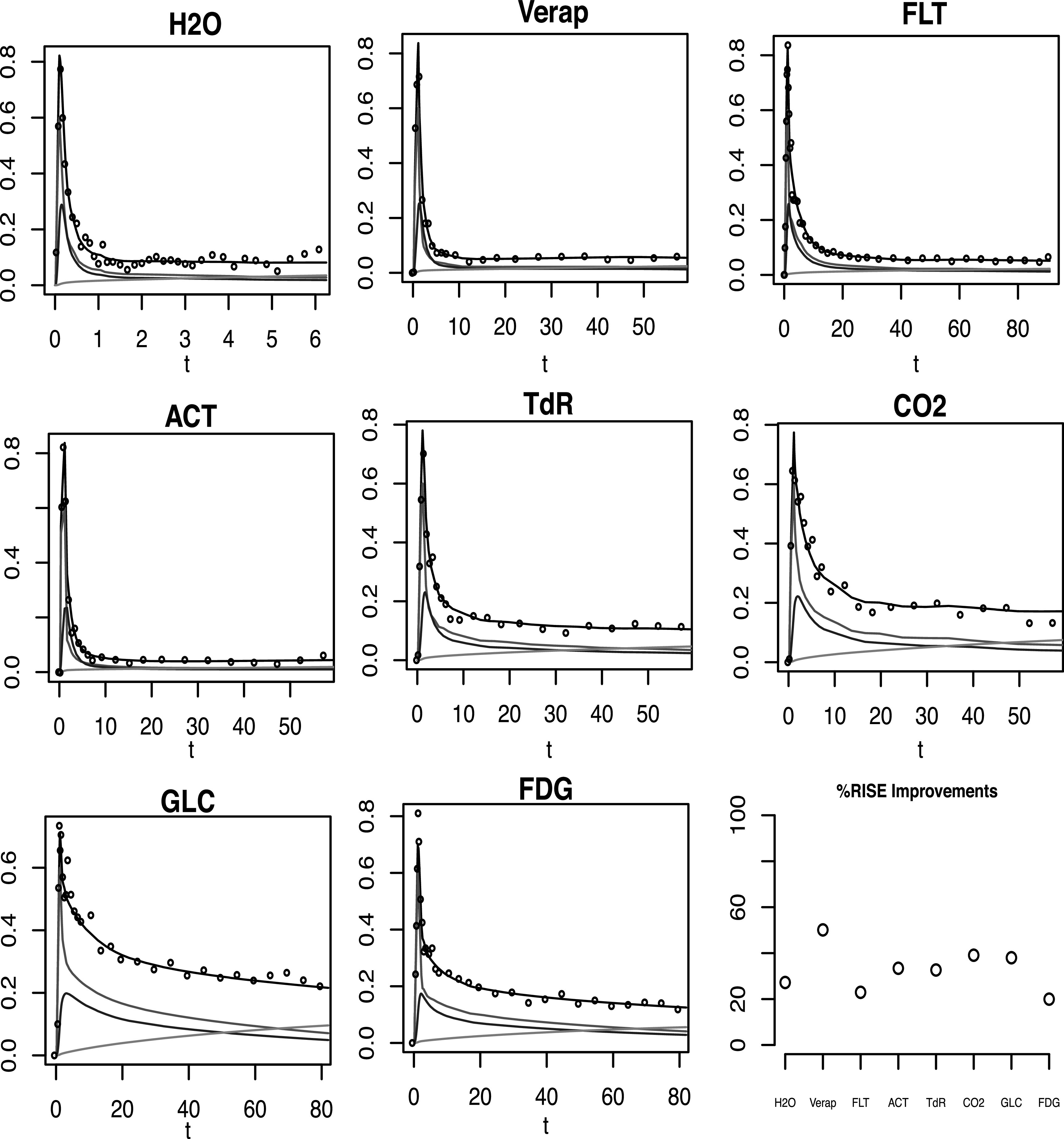
Sample ROI data (black dots) for a simulation corresponding to an intermediate dispersion (blue) and spillover (green) - *p*_*d*_ = 0.4; *p*_*b*_ = 0.75 in equations (15), (16). Lower right shows the average percent improvement in the RMISE of the AIF using the regularised extraction method versus direct use of the ROI data.

\begin{eqnarray*}{z}_{i}={C}_{T}({t}_{i})+{\sigma }_{i}{\epsilon }_{i}\,\,,\,\,i=1,2,...,n,\end{eqnarray*}

where {*ϵ*_*i*_, *i* = 1, 2,...,*n*} is a simulated random sample from a *N*(0, 1) distribution. Sample data for and intermediate case are shown for each tracer in figure 5. While the noise level in these experiments is somewhat low, if anything this facilitates performance of the direct approach when the data is contamination free.

**Figure 6.**
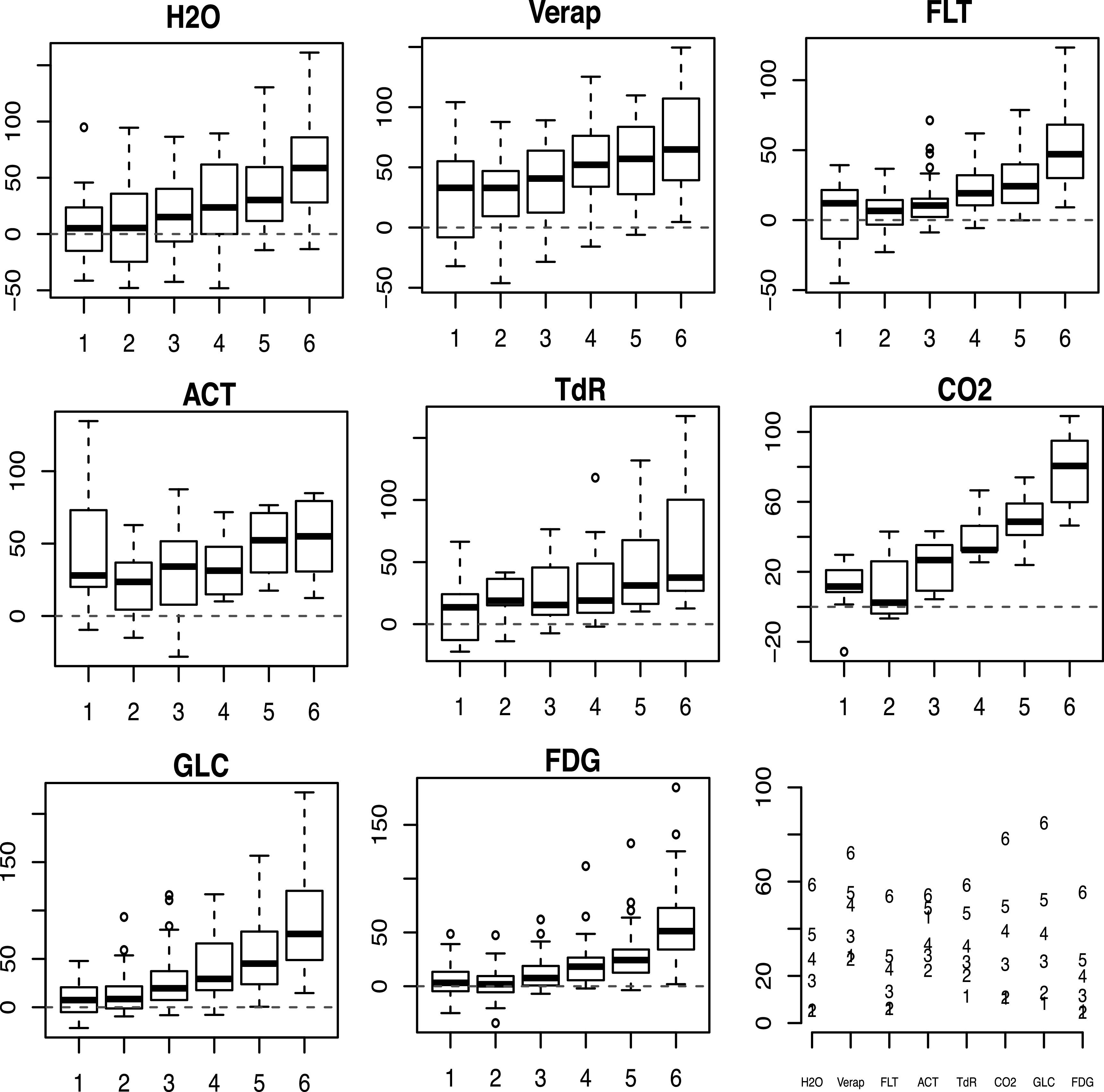
Boxplots of percent improvements in the RMISE of the AIF error for different tracers and varying amounts of dispersion and spillover. The numbers [**1-6**] correspond to - (*p*_*d*_, *p*_*b*_) = (0.1, 0.125); (0.2, 0.25); (0.3, 0.5); (0.4, 0.75); (0.5, 1); (0.6, 2) in equation (15). Red dashed lines corresponds to 0% improvement. Lower right shows average RMISE values across simulations conducted for each tracer and each of the six levels (shown with digits) of dispersion and spillover.

AIF estimates were evaluated for each simulated time-course using the proposed constrained regularised extraction method and also directly using the raw time-course. Estimated AIFs were scaled using a simulated measurement of the true AIF *C*_*p*_ at a single time-point—2 min for H2O, 10 minutes for FDG and GLC, and 5 min for other tracers. The average of the square-root of the in figure 6. With contamination free data, the direct method clearly out-performs the regularised extraction procedure. However in the presence of contamination, the reliability of the direct method deteriorates and the constrained regularisation procedure is much preferred.

## 5. Discussion

Linear representation for the AIF in terms of an impulse response to the injection profile is explored as a basis for image-based AIF extraction in PET. The formulation facilitates specification of constraints needed to address the inherent non-identifiability associated with AIF extraction from blood-pool ROI data that may be contaminated by the presence of spillover from background and/or dispersion. A fully data-adaptive regularised AIF extraction procedure is developed. The practical significance of the technique is illustrated by application to PET brain studies involving a range tracers that have been used in imaging cancer. For the specific examples considered for image-based extraction, the input ROI data used are collections of segmentation-derived time-courses. In this context the ability to pool AIF estimates from the analysis of separate ROIs is important. The numerical studies presented show significant improvements achieved by the AIF extraction method even when quite limited amounts of contamination are present.

An advantage of the impulse response approach is that it gives the ability to easily adapt to cases where the injection protocol might have deviated from an idealised bolus or square-wave pattern. This feature gives potential to better represent the true AIF in such cases with a consequent impact on the accuracy of kinetic information derived using the AIF. The convolution of the population impulse response function, defined by ***μ***_*θ*_ in equation (8), and the injection wave-form also provides a new way to construct more adaptable alternatives to template-based AIF extraction methods such as (Olshen and O’Sullivan 1997, Christensen *et al* 2014, Rissanen *et al* 2015).

An important part of the proposed methodology is that is gives the ability to combine information about the AIF from a collection of separate ROIs. This has a similarity to the techniques described in (Feng *et al* 1997, Wong *et al* 2001, 2002) for analysis of voxel-level of dynamic FDG data in the brain. While the latter works focus on direct parametric representation of the AIF, it may be that the approach could also be adapted, using a constrained impulse response formulation to provide more flexibility and stability.

In general, unless the ROI data is known to be free of contamination, the estimated AIF requires scaling. Some alternative schemes for scaling are suggested but in the context of the brain imaging examples considered, the most quantitatively satisfactory approach is to scale the AIF using a suitable directly measured blood sample. Further development of scaling methods would be valuable. In this context there may also be a possibility to modify techniques in (Xiu *et al* 2023) to the impulse response setting.

Adaptation of the impulse response methodology to small animal studies would be worthwhile. This could supplement current techniques (Laforest *et al* 2005, Lanz *et al* 2014, Meyer *et al* 2017). But since circulatory dynamics in small animals is not the same as in humans, the development of a statistical characterisation of a target tracer impulse response would need to be based on directly sampled blood data for the animal. In this context recently described experimental techniques for direct measurement of arterial time-courses in small animals, e.g. (Mann *et al* 2019), could be essential. A successful adaptation to small animal PET setting could facilitate more sophistication in the type of kinetic information that might be routinely recovered from pre-clinical PET studies. The proposed approach may also have some potential in the context of dynamic tracer studies with MR (Huang *et al* 2019) and CT (Wang *et al* 2018).

**Figure 7.**
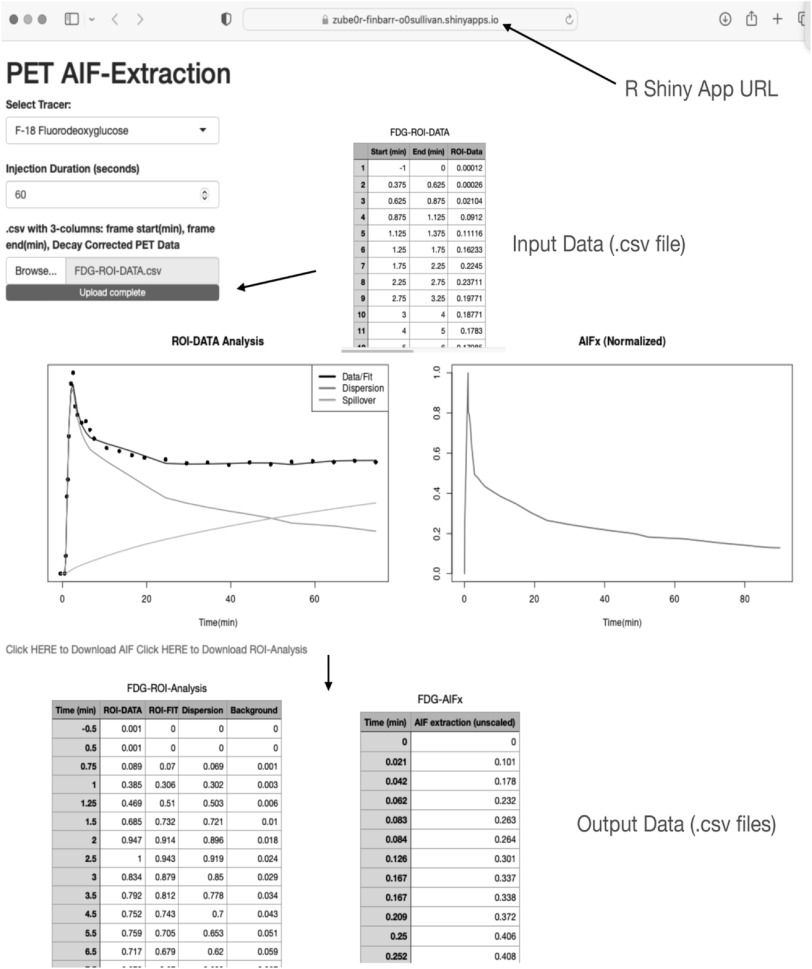
R Shiny App interface for implementation for the AIF extraction method based on ROI data that may be impacted by background and/or dispersion effects. In the App, the user specifies the tracer, the duration for the tracer injection. PET measured time-course data for a suitable ROI must be provided in. csv file. Results of the extraction process are provided graphically by the App and the associated data are available for download into two. csv files. The URL for the App is: https://zube0r-finbarr-o0sullivan.shinyapps.io/xaif-app/.

The methodology described in this paper has been implemented as a freely available R Shiny (Chang *et al* 2023) application hosted on a web-accessible server by ShinyApps.io. Figure 7 in appendix B gives information on the appendix Reasonable requests for assistance in the use of the methods described will be facilitated.

## Data Availability

The data that support the findings of this study will be openly available following an embargo at the following URL/DOI: https://doi.org/https://github.com/fos-ucc/PMB-2023-AIF-Extraction. Data will be available from 3 April 2024.

## Appendix A. Formal non-identifiability of the impulse response in the presence of dispersion and/or background spillover

Theorem 1. Consider the determination of the normalised impulse response, ${ \mathcal R }$, based on the model in equation (3) when data are continuously sampled, noise-free and the delay Δ parameter is known. Assume the unknown impulse response and the injection profile, *C*_*I*_, are both continuous and have finite moments of all orders, i.e. ${\int }_{0}^{\infty }{ \mathcal R }(t){t}^{k}{dt}\lt \infty $ for all $k\geqslant 0$. Convolution with *C*_*I*_ is assumed to be (uniquely) invertible. In addition assume that for some times ${T}_{a},{T}_{b}\in [0,T]$, where ${ \mathcal R }({T}_{a}),{ \mathcal R }({T}_{b}),\tilde{S}({T}_{a})$ and $\tilde{S}({T}_{b})$ are strictly positive and that the impulse response contrast ratio, ${ \mathcal R }({T}_{b})/{ \mathcal R }({T}_{a})$, does not match the corresponding ratio, $\tilde{S}({T}_{b})/\tilde{S}({T}_{a})$, for $\tilde{S}$.

(i) If ${\alpha }_{2}={\alpha }_{3}=0$ (no dispersion/spillover) the measured data uniquely determines ${ \mathcal R }$
(ii) If ${\alpha }_{2}\gt 0,\phi \gt 0,{\alpha }_{1}={\alpha }_{3}=0$ (pure dispersion), there is not a unique ${ \mathcal R }$ that matches the measured data. However if the value of integral, ${\mu }_{1}={\int }_{0}^{\infty }t{ \mathcal R }(t){dt}$, is also known, then ${ \mathcal R }$ is uniquely determined by the data.
(iii) If ${\alpha }_{3}\gt 0,{\alpha }_{2}=0,{\alpha }_{1}\gt 0$ (pure spillover contamination) the value of ${ \mathcal R }$ that matches the data is not unique. However if the contrast ratio ${\mu }_{2}={ \mathcal R }({T}_{b})/{ \mathcal R }({T}_{a})$ is also known, then ${ \mathcal R }$ is uniquely determined by the data.

Proof. To simplify notation, the delay is assumed to be zero, i.e. ${\mathrm{\Delta }}=0$. Since convolution with *C*_*I*_ is uniquely invertible (for example, *C*_*I*_, cannot be identically zero), by using Fourier transforms the continuously sampled noise free data *z*_*t*_ can be mapped to a function ${y}_{t}={{ \mathcal F }}^{-1}\left(\hat{z}(\xi )/{\hat{C}}_{I}(\xi )\right)$ where ${ \mathcal F }$ is the 1d normalised Fourier transform and $\hat{z}$ and ${\hat{C}}_{I}$ are the Fourier transforms of *z* and *C*_*I*_. With this transformation, the available continuous noise-free data arising from equation (3) is equivalent to the data *y*_*t*_ where

\begin{eqnarray*}{y}_{t}={\alpha }_{1}{ \mathcal R }(t)+{\alpha }_{2}{E}_{\phi }\,\ast \,{ \mathcal R }[t]+{\alpha }_{3}\tilde{S}(t)\end{eqnarray*}

i.e. ${ \mathcal R }$ is identifiably based on the data *z* in equation (3) if and only if, it is identifiable based on the data *y* in equation (17).

(i) With ${\alpha }_{2}={\alpha }_{3}=0$, the measured data is directly proportional to ${ \mathcal R }$ so the normalised impulse response is uniquely determined.
(ii) When $\phi \gt 0$, direct Fourier analysis shows that for any $0\lt \phi ^{\prime} \lt \phi $ with $A=\tfrac{\phi ^{\prime} }{\phi }$ and $B=1-\tfrac{\phi ^{\prime} }{\phi }$, the exponential density *E*_*ϕ*_, ${E}_{\phi }(t)=\tfrac{1}{\phi }{e}^{-t/\phi }$, can be expressed as the sum: ${{AE}}_{\phi ^{\prime} }+{{BE}}_{\phi ^{\prime} }\,\ast \,{E}_{\phi }$. This allows exponential convolution with a possible ${ \mathcal R }$ to be equivalently expressed in terms of a different exponential density and IR - ${E}_{\phi ^{\prime} }$ and ${ \mathcal R }^{\prime} $
  \begin{eqnarray*}{E}_{\phi }={{AE}}_{\phi ^{\prime} }+{{BE}}_{\phi ^{\prime} }\,\ast \,{E}_{\phi }\Longrightarrow {E}_{\phi }\,\ast \,{ \mathcal R }={E}_{\phi ^{\prime} }\,\ast \,(A{ \mathcal R }+{{BE}}_{\phi }\,\ast \,{ \mathcal R })={E}_{\phi ^{\prime} }\,\ast \,{ \mathcal R }^{\prime} \end{eqnarray*}
  Thus with pure dispersive contamination (${\alpha }_{3}=0$, ${\alpha }_{1}=0$, ${\alpha }_{2}\gt 0$ and $\phi \gt 0$), by letting ${ \mathcal R }^{\prime} =A{ \mathcal R }+{{BE}}_{\phi }\,\ast \,{ \mathcal R }$ it is clear that ${\int }_{0}^{\infty }{ \mathcal R }^{\prime} (t){dt}=A\,+\,B\,=\,1$ and ${\alpha }_{2}{E}_{\phi }\,\ast \,{ \mathcal R }={\alpha }_{2}{E}_{\phi ^{\prime} }\,\ast \,{ \mathcal R }^{\prime} $. But since ${ \mathcal R }^{\prime} \ne { \mathcal R }$ unless $\phi ^{\prime} =0$, there are different choices for the impulse response corresponding to same the data. If ${\int }_{0}^{\infty }t{ \mathcal R }^{\prime} (t){dt}={\int }_{0}^{\infty }t{ \mathcal R }(t){dt}={\mu }_{1}$, it is easy to check that we must have $\phi ^{\prime} =0$ so ${ \mathcal R }^{\prime} ={ \mathcal R }$, guaranteeing uniqueness of the impulse response under the assumed constraint.
(iii) For pure spillover contamination (${\alpha }_{1}\gt 0$, ${\alpha }_{2}=0$ and ${\alpha }_{3}\gt 0$), let $\gamma ={\alpha }_{3}/{\alpha }_{1}$. For any $0\lt p\lt 1$ notice that

\begin{eqnarray*}{\alpha }_{1}{ \mathcal R }+{\alpha }_{3}\tilde{S}={\alpha }_{1}[{ \mathcal R }+\gamma \tilde{S}]={\alpha }_{1}[{ \mathcal R }+p\gamma \tilde{S}+(1-p)\gamma \tilde{S}]+(1-p)\gamma \tilde{S}]\equiv {\alpha }_{1}^{\prime} { \mathcal R }^{\prime} +{\alpha }_{3}^{\prime} \tilde{S}\end{eqnarray*}

where ${\alpha }_{1}^{\prime} ={\alpha }_{1}[1+p\gamma ]$, ${ \mathcal R }^{\prime} =({ \mathcal R }+p\gamma \tilde{S})/(1+p\gamma )$ and ${\alpha }_{3}^{\prime} ={\alpha }_{3}(1-p)$. ${ \mathcal R }^{\prime} $ is non-negative and integrates to unity. Since ${ \mathcal R }^{\prime} \ne { \mathcal R }$ for any choice of *p*, $0\lt p\lt 1$, there is not a unique choice of ${ \mathcal R }$ corresponding to the data. However, if the contrast ratio is constrained by the known value ${\mu }_{2}=\tfrac{{ \mathcal R }({T}_{b})}{{ \mathcal R }({T}_{a})}$ then

\begin{eqnarray*}\displaystyle \frac{{ \mathcal R }({T}_{b})}{{ \mathcal R }({T}_{a})}=\displaystyle \frac{{ \mathcal R }^{\prime} ({T}_{b})}{{ \mathcal R }^{\prime} ({T}_{a})}=\displaystyle \frac{{ \mathcal R }({T}_{b})+p\gamma \tilde{S}({T}_{b})}{{ \mathcal R }({T}_{a})+p\gamma \tilde{S}({T}_{a})}\end{eqnarray*}

But since $\tfrac{\tilde{S}({T}_{b})}{\tilde{S}({T}_{a})}\ne \tfrac{{ \mathcal R }({T}_{b})}{{ \mathcal R }({T}_{a})}$ and $\gamma \gt 0$, equality above requires *p* = 0. This implies ${ \mathcal R }^{\prime} ={ \mathcal R }$ and hence the contrast ratio constraint resolves non-identifiability in this case. □
